# Cutaneous Medicine

**Published:** 1896-01

**Authors:** 


### Book Reviews.

Cutaneous Medicine. A Systematic Treatise on the Diseases of the Skin. By Louis A. Duhring, M. D., Professor of Diseases of the Skin in the University of Pennsylvania, etc. Octavo, pp. vii.—221. Illustrated. Philadelphia: J. B. Lippincott Company. 1895.

Dr. Duhring's reputation and the standard character of his treatise on diseases of the skin renders the announcement of his latest work on cutaneous medicine of more than ordinary interest. Part I. of this work is now before us and comes up fully to our expectations. The author is nothing if not practical, and possesses in a high degree the art of bringing out the principles and practical bearing of the relations of the subject in question.

The sections on anatomy and physiology, which come first, and the understanding of which are so necessary to the successful diagnosis and treatment, are presented in a style which divests them of anything like dryness and makes them both satisfactory and instructive. The sections are illustrated by admirably executed drawings that embody the latest researches made in the morphological histology of cutaneous tissues.

It is pertinent to say that the important subject of the relationship of general diseases and conditions to the local skin affections is discussed at length, and in a manner which appeals to the general practitioner, to whom it is so important, as well as to the specialist.

A feature which will be appreciated by the student is the clearness and conciseness of description. The author has avoided those minute unnecessary points which so often tend to obscure rather than to make clear the subject. A careful examination of the treatise convinces us that he has accomplished his task with painstaking fidelity, representing the most advanced scientific and clinical position of the art. E. W.

